# Supplementary material for: Xanomeline displays concomitant orthosteric and allosteric binding modes at the M4 mAChR
Source: Nat Commun. 2023 Sep 6;14:5440. doi: 10.1038/s41467-023-41199-5 (PMC10482975; doi:10.1038/s41467-023-41199-5)
Supplement: Supplementary file 1 — Supplementary Information [file 41467_2023_41199_MOESM1_ESM.pdf]

Supplemental Information

Xanomeline displays concomitant orthosteric and allosteric binding modes at the M_4 mAChR

Wessel A.C. Burger[1,2,*], Vi Pham[1,*], Ziva Vuckovic[1,*], Alexander S. Powers[3,4*], Jesse I. Mobbs[1,2], Yianni Laloudakis[3], Alisa Glukhova[1,2], Denise Wootten[1,2], Andrew B. Tobin[5], Patrick M. Sexton[1,2], Steven M. Paul[6], Christian C. Felder[6], Radostin Danev[7], Ron O. Dror[4,#], Arthur Christopoulos[1,2,8,#], Celine Valant[1,#], and David M. Thal[1,2, #]

1 Drug Discovery Biology, Monash Institute of Pharmaceutical Sciences, Monash University, Parkville, VIC 3052, Australia.

2 Australian Research Council Centre for Cryo-Electron Microscopy of Membrane Proteins, Monash Institute of Pharmaceutical Sciences, Monash University, Parkville, VIC 3052, Australia.

3 Department of Chemistry, Stanford University, Stanford, CA 94305, USA.

4 Departments of Computer Science, Structural Biology, and Molecular and Cellular Physiology, Stanford University, Stanford, CA 94305, USA.

5 The Advanced Research Centre (ARC), School of Biomolecular Sciences, College of Medical, Veterinary and Life Sciences, University of Glasgow, Glasgow G12 8QQ, United Kingdom.

6 Karuna Therapeutics, Boston, Massachusetts 02110, United States.

7 Graduate School of Medicine, University of Tokyo, N415, 7-3-1 Hongo, Bunkyo-ku, 113-0033 Tokyo, Japan.

8 Neuromedicines Discovery Centre, Monash University, Parkville, VIC 3052, Australia.

* These authors contributed equally.

[#] Correspondence: ron.dror@stanford.edu (R.O.D.); arthur.christopoulos@monash.edu (A.C.); celine.valant@monash.edu (C.V.); david.thal@monash.edu (D.M.T.)

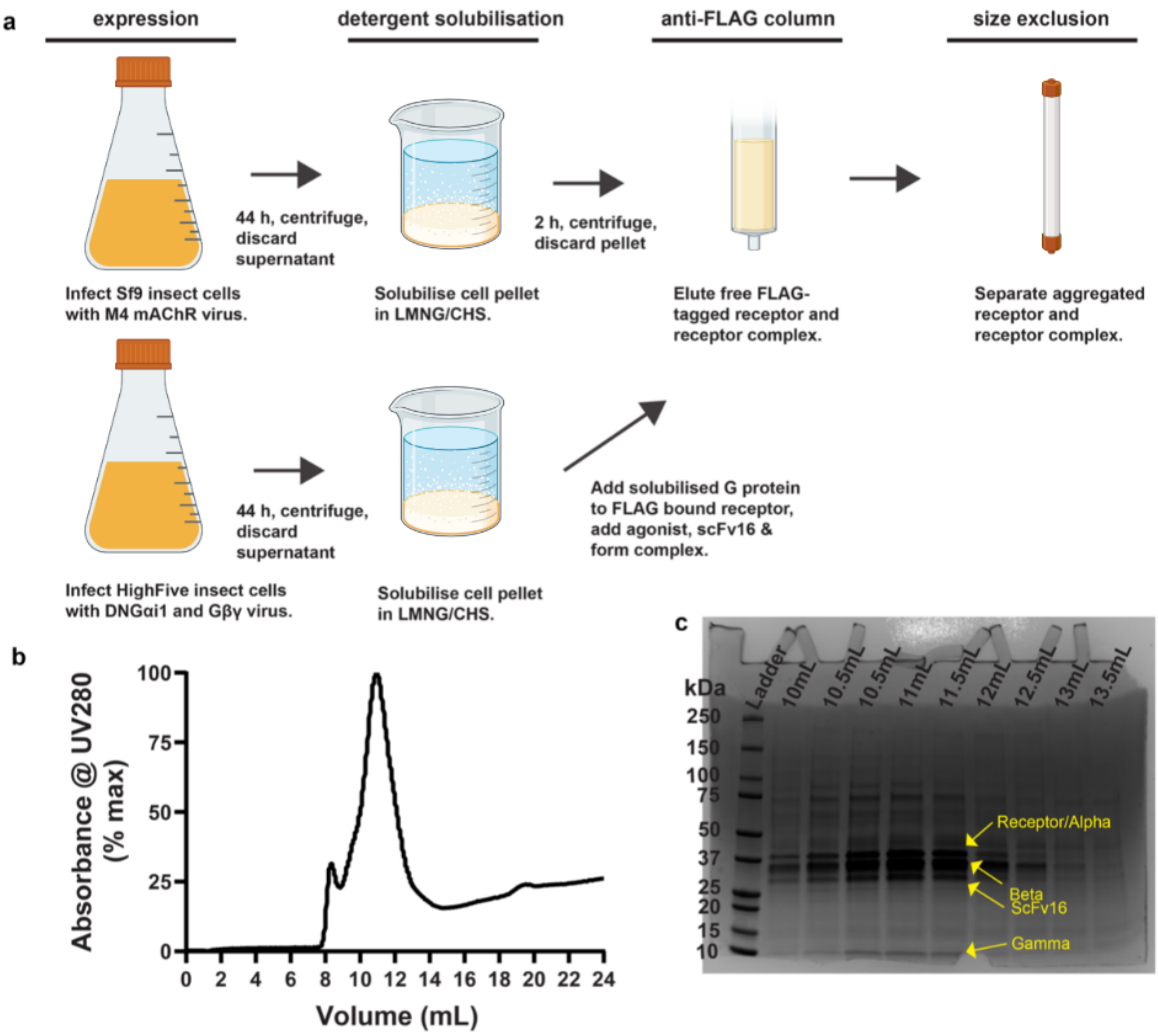

Supplementary Figure 1. Purification of the xano-M_4R-G_i complex. (a) Purification flow chart of the xano-M_4R-G_i complex. **(b)** Elution profile of the xano-M_4R-G_i complex in size exclusion chromatography using a Superdex 200 Increase 10/30 and **(c)** Coomassie staining of SDS-PAGE gel (uncropped) with the corresponding SEC fractions. Created with BioRender.com.

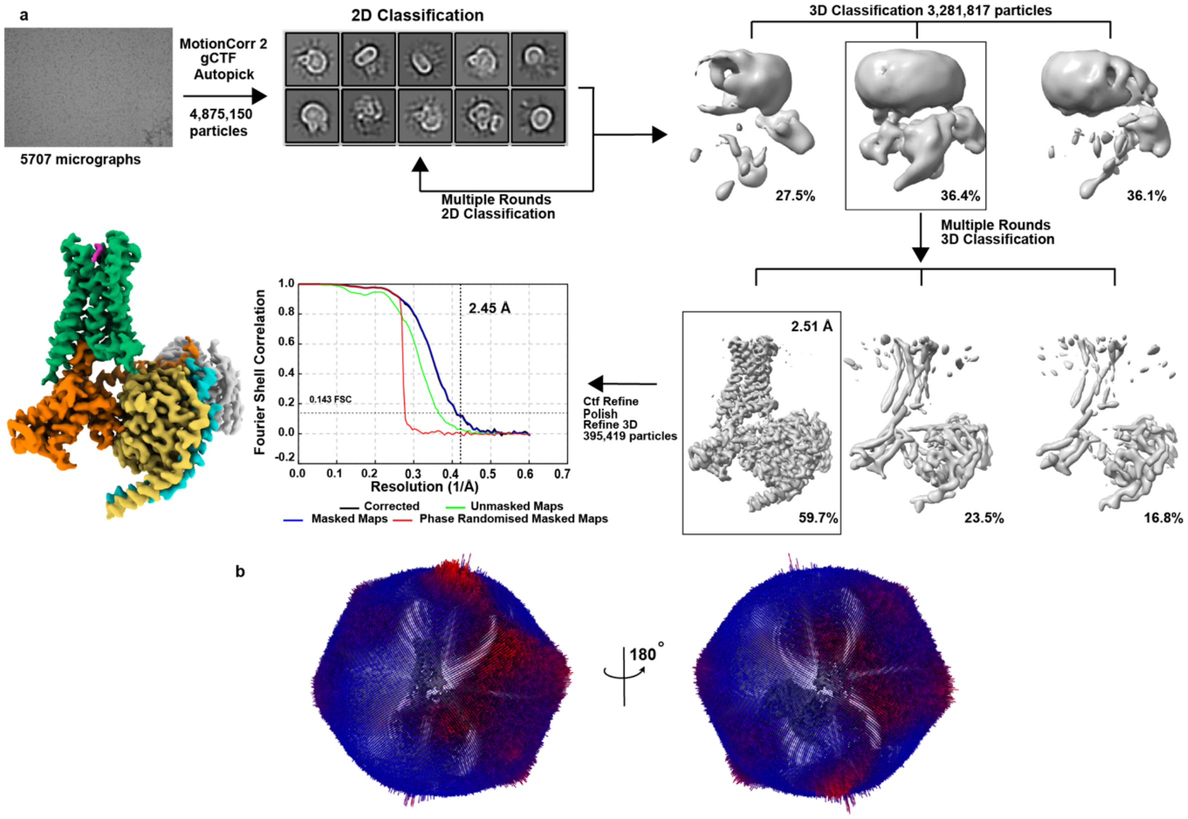

Supplementary Figure 2. Cryo-EM data processing for the xano-M$_4$R-G$_i$ complex. (a) Flow chart for cryo-EM analysis as described in methods leading to final cryo-EM density maps at a resolution of 2.45 Å. **(b)** Angular distribution of particles projections overlayed onto final map.

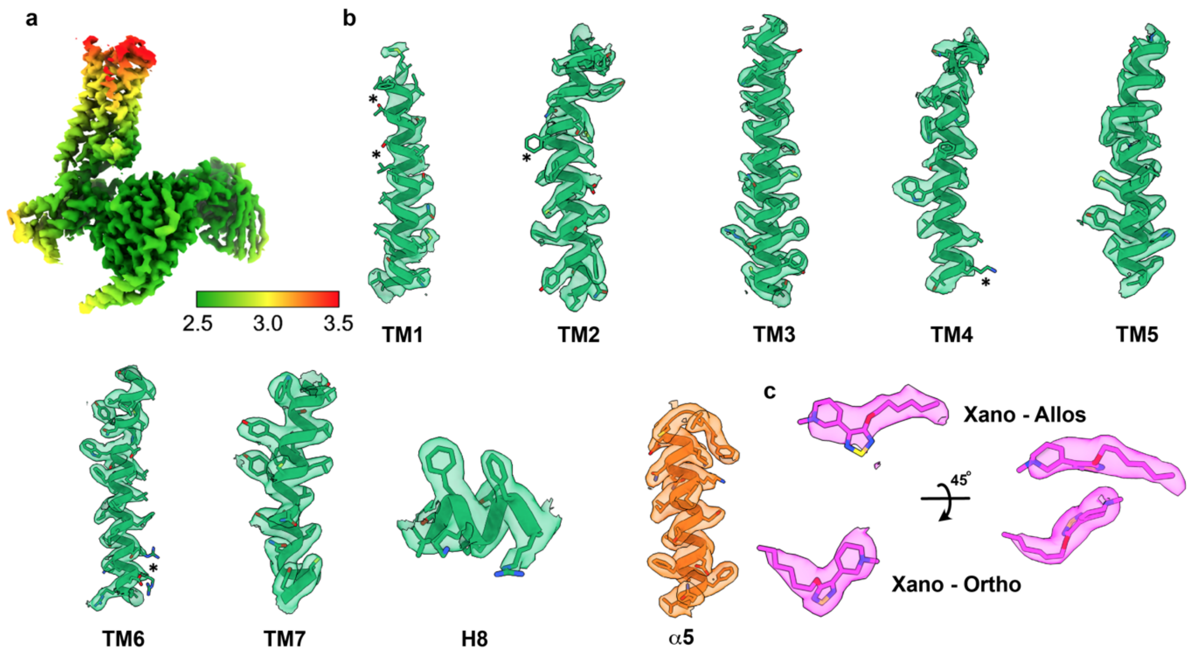

Supplementary Figure 3. Cryo-EM density maps. (a) Local resolution map of the xano-M_4R-G_i complex calculated in RELION 3.1. **(b)** Shown as cartoons are representative regions of the transmembrane domains of the M_4 mAChR and the α5 of G_{i1} modeled into the cryo-EM map, which is shown as a transparent surface contoured at 0.026. **(c)** Shown in stick representation coloured by heteroatom are xanomeline as found in the orthosteric and allosteric sites with two different points of view. The cryo-EM map is shown as a transparent surface contoured at 0.026. Some residues were not well-supported with EM density beyond the β-carbon and were left fully modelled (labelled with *).

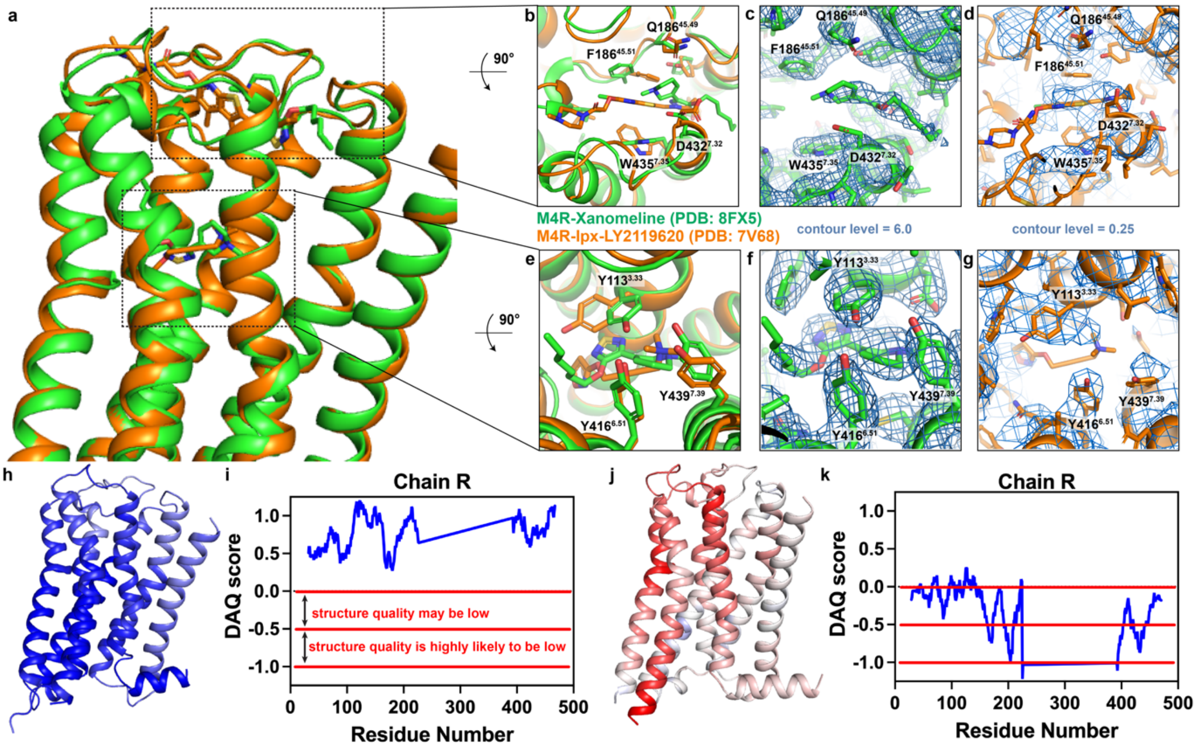

Supplementary Figure 4. Comparison of M$_4$ mAChR cryo-EM structures. (a) Comparison of the xano-M$_4$R-G$_{i1}$ complex (green) and the LY2119620-ipx-M$_4$R-G$_{i1}$ complex (PDB: 7V68, orange). **(b)** View of the allosteric site comparing the position of key allosteric residues F186 and W435. Comparison of EM density of the corresponding allosteric binding site residues from **(c)** the xano-M$_4$R-G$_{i1}$ complex at a map contour of 6.0 and **(d)** the LY2119620-ipx-M$_4$R-G$_{i1}$ complex at a map contour of 0.25. **(e)** View of the orthosteric site comparing the position of tyrosine lid residues Y113, Y416, and Y439. Comparison of EM density of the orthosteric binding site residues of **(f)** the xano-M$_4$R-G$_{i1}$ complex at a map contour of 6.0 and **(g)** the LY2119620-ipx-M$_4$R-G$_{i1}$ complex at a map contour of 0.25. **(h-k)** A Deep-learning-based residue-wise Quality Assessment score for cryo-EM models (DAQ-score) of **(h,i)** the xano-M$_4$R-G$_{i1}$ complex and **(j,k)** the LY2119620-ipx-M$_4$R-G$_{i1}$ complex. A DAQ-score that is positive (coloured blue at values of 1) indicates a correct assignment. A DAQ-score near 0 (coloured white) indicates a position in the map that lacks a distinct density pattern for the assigned amino acid. DAQ-scores less than 0 (coloured red at -1) indicate a position that could be misassigned or poorly fit. **(h,j)** DAQ scores mapped onto the corresponding receptor model by colour. **(i,k)** DAQ scores plotted residue by residue each Cα atom.

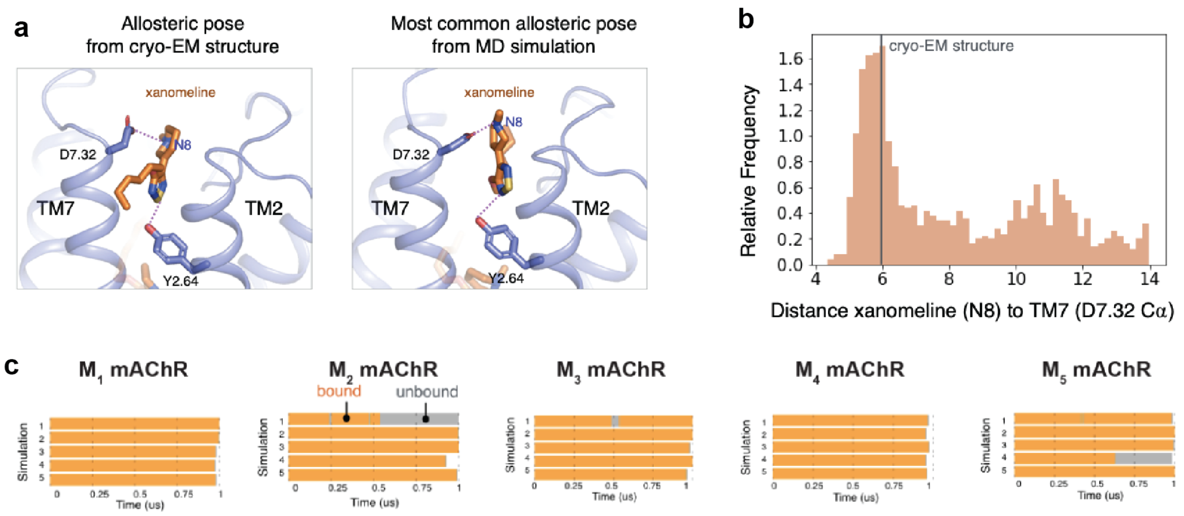

Supplementary Figure 5. Xanomeline binding to the allosteric site of mAChRs in MD simulations. (a) In MD simulations of spontaneous binding to the allosteric site, xanomeline occupies a similar location to that observed in the cryo-EM structure, forming interactions with TM7 and TM2. A representative snapshot of the most common pose from MD simulations is shown at right in comparison to the cryo-EM structure at left. **(b)** The orientation of the xanomeline molecule with respect to the allosteric site was quantified by measuring the distance between xanomeline's ternary amine (N8) to the Cα of residue $D432^{7.32}$ on TM7. The histogram is over simulation frames from five independent simulations of spontaneous binding (specifically, all frames in which xanomeline is bound to the allosteric site; see Methods). The most frequent distance in simulation matches the distance observed in the cryo-EM structure (vertical grey line). **(c)** In MD simulations, xanomeline stays bound to the allosteric site at the remaining mAChR subtypes for similar durations. A xanomeline molecule was placed into the orthosteric and allosteric binding site, similar to the poses observed in the cryo-EM structure, and simulations then initiated to measure the duration of binding of xanomeline at the allosteric site. Each horizontal bar represents an independent simulation, and shows when the allosteric site is vacant (grey) or occupied (orange) by xanomeline (see Methods).

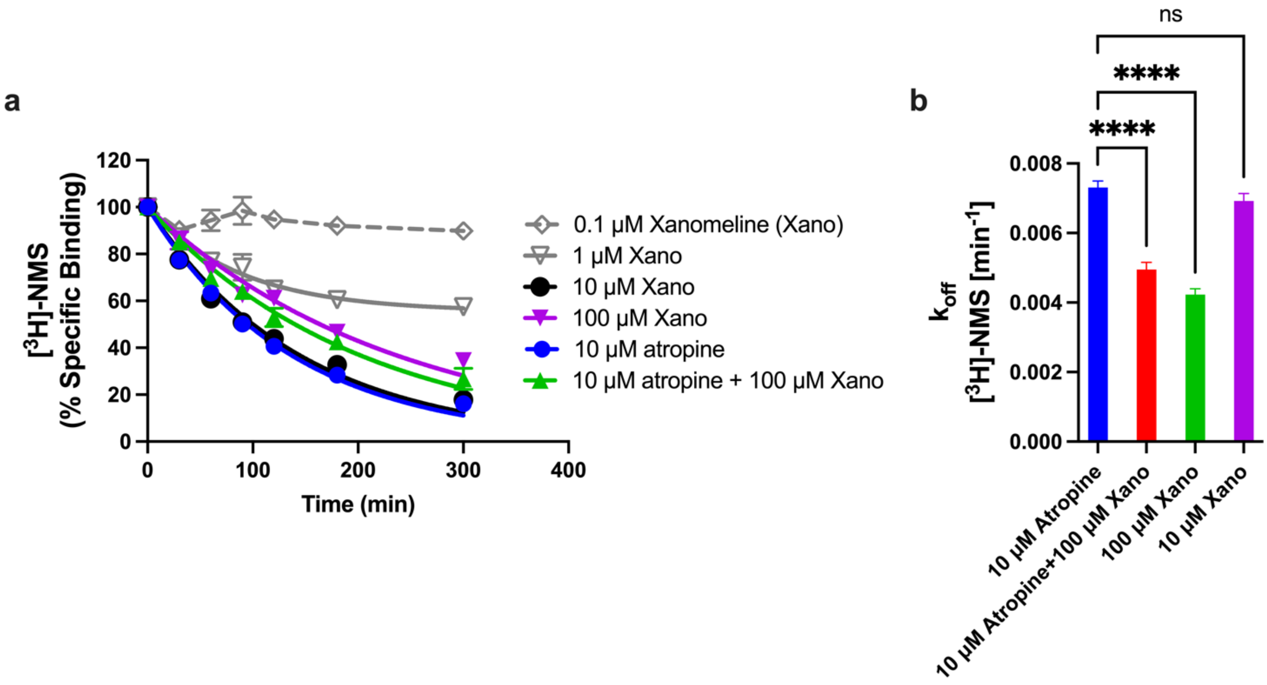

Supplementary Figure 6. Xanomeline binds to both the orthosteric and allosteric site in [^{3}H]-NMS dissociation experiments (a) [^{3}H]-NMS dissociation via isotopic dilution with increasing concentrations of xanomeline alone, atropine (10 µM) alone, or the combination of atropine (10 µM) and xanomeline (100 µM). Data points represent the mean ± S.E.M. of four individual experiments performed in duplicate. Data were fitted to a one-phase exponential decay model with the exception of the binding observed in the presence of the lowest concentration of xanomeline (0.1 µm). **(b)** [^{3}H]-NMS dissociation rate constants (k_{off}; min^{-1}) derived from the indicated experiments in panel (A). **** denotes k_{off} values that are statistically significantly different from the control value determined in the presence of 10 µM atropine ($p < 0.0001$; one-way ANOVA followed by Dunnett's multiple comparison test).

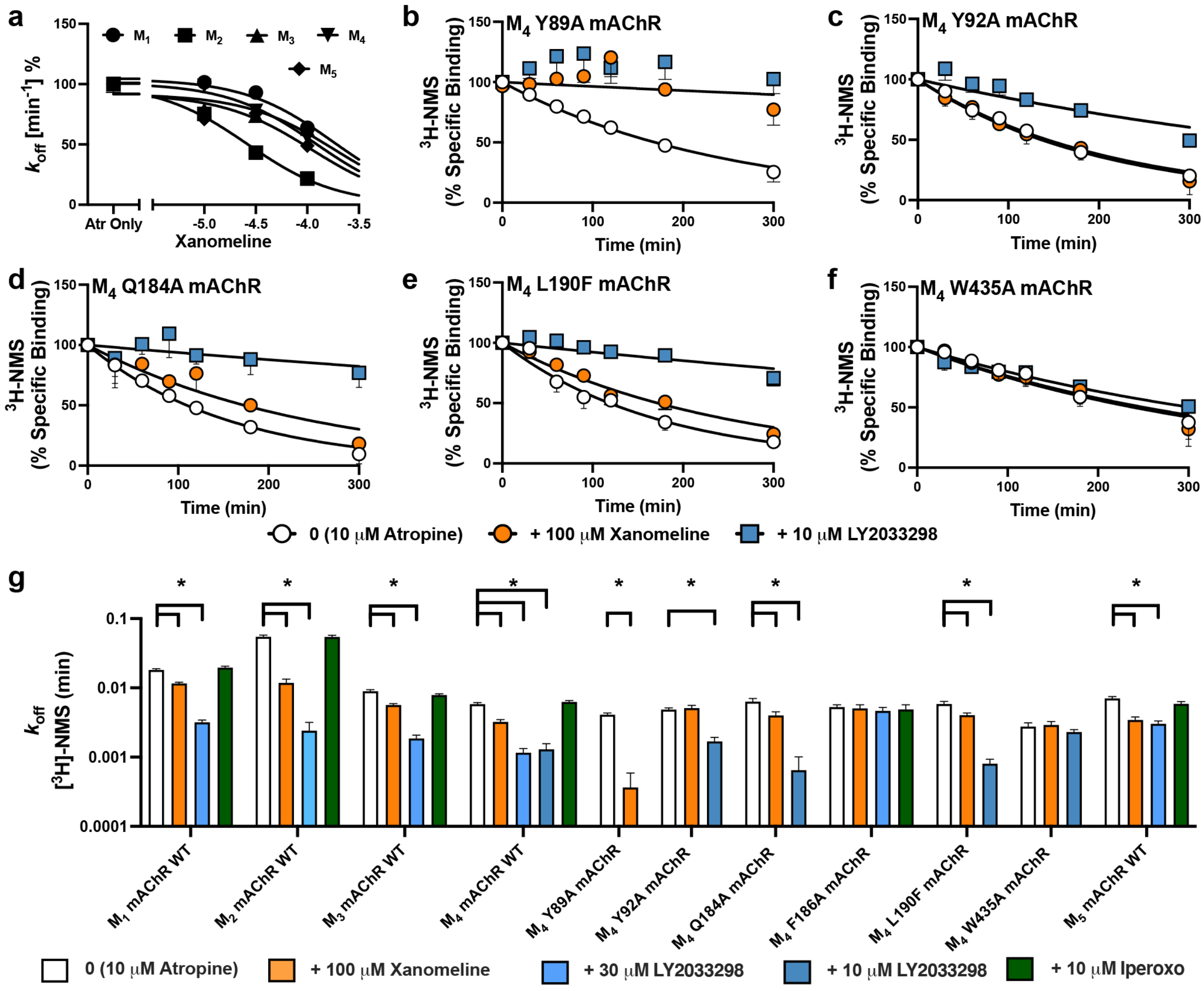

Supplementary Figure 7. [^{3}H]-NMS dissociation experiments at all mAChR subtypes and M_4 mAChR point mutants. (a) Dissociation rate constants (k_{off}; min^{-1}) of [^{3}H]-NMS as a function of xanomeline concentration. Data represent the mean ± S.E.M. of five to twelve individual experiments performed in duplicate, M_1 mAChR; n = 6, M_2 mAChR; n = 10, M_3 mAChR; n = 7, M_4 mAChR; n = 12, M_5 mAChR; n = 5. Curves represent the global fit of a three-parameter logistic equation to the data points. **(c-f)** [^{3}H]-NMS dissociation via isotopic dilution with 10 µM atropine alone (0), or in the presence (+), of 100 µM xanomeline or 10 µM LY2033298 at the **(b)** M_4 Y89A mAChR, **(c)** M_4 Y92A mAChR, **(d)** M_4 Q184A mAChR, **(e)** M_4 L190F mAChR, **(f)** M_4 W435A mAChR mutants. Data points represent the mean ± S.E.M. of four to five individual experiments performed in duplicate. For **(b-f)**, 10 µM atropine alone & + 100 µM xanomeline n = 5, + 10 µM LY2033298 n = 4. Data were fitted to a one-phase exponential decay model. **(g)** Dissociation rate constants (k_{off}; min^{-1}) of [^{3}H]-NMS in the presence of 10 µM atropine alone (0) or in combination (+) with 10 µM iperoxo, 10 µM LY2033209, 30 µM LY2033298 or 100 µM xanomeline at all characterised WT and mutant mAChRs. M_1 mAChR; 10 µM atropine alone & + 10 µM iperoxo & + 100 µM xanomeline n = 6, + 30 µM LY2033298 n = 3. M_2 mAChR; 10 µM atropine alone n = 10, + 10 µM iperoxo & + 30 µM LY2033298 & + 100 µM xanomeline n = 6. M_3 mAChR; 10 µM atropine alone & + 10 µM iperoxo & + 100 µM xanomeline n = 7, + 30 µM LY2033298 n = 6. M_4 mAChR wild type**;** 10 µM atropine alone n = 14, + 10 µM iperoxo n = 5, + 30 µM LY2033298 n = 7, + 10 µM LY2033298 n = 4, + 100 µM xanomeline n = 13. M_4 Y89A, Y92A, Q184A, L190F, W435A mAChR; 10 µM atropine alone & + 100 µM xanomeline n

= 5, + 10 µM LY2033298 *n* = 4. M_4 F186^{ECL2}A; 10 µM atropine alone *n* = 4, + 10 µM iperoxo & + 30 µM LY2033298 & + 100 µM xanomeline *n* = 3. M_5 mAChR; 10 µM atropine alone & + 10 µM iperoxo & + 100 µM xanomeline *n* = 5, + 30 µM LY2033298 *n* = 4. * denotes values that were statistically significantly different from the control value determined in the presence of 10 µM atropine alone ($p < 0.05$; one-way ANOVA and Dunnett's multiple comparison test).

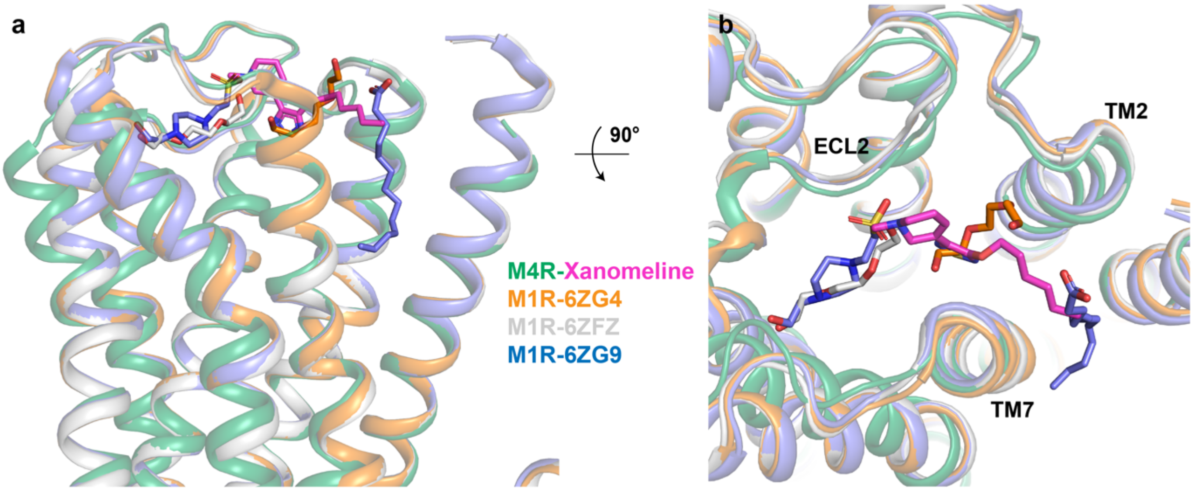

Supplementary Figure 8. **Comparison of M_1 and M_4 mAChR allosteric sites.** Comparison of the xanomeline (magenta sticks) bound allosteric site at the M_4 mAChR to the allosteric site of the recent M_1 mAChR structures (PDB: 6ZG4, 6ZFZ, 6ZG9) where polyethylene glycol (PEG) (orange and grey sticks), HEPES (blue sticks) or oleic acid (blue sticks) molecules were observed bound in, and around, the allosteric binding site.

Supplementary Table 1 | Cryo-EM data collection and refinement statistics

**Data Collection**	**$M_{4\Delta ICL3}$mAChR/dnG_{i1}/scFv16/Xanomeline**
**Micrographs**	5707
**Electron dose (e^-/Å^2)**	0.738
**Voltage (kV)**	300
**Pixel size (Å)**	0.83
**Defocus range (μM)**	0.5-1.5
**Symmetry Imposed**	C1
**Particles (final map)**	395419
**Resolution (0.143 FSC) (Å)**	2.45
**Refinement**	
**CCmap_model**	0.90
**Map Sharpening B factor (Å^2)**	-10
**Model Quality**	
**R.m.s deviations**	
**Bond Length (Å)**	0.004
**Bond angles (°)**	0.824
**Ramachandran**	
**Favoured (%)**	98.02
**Outliers (%)**	0.00
**Rotamer outliers (%)**	0.11
**C-Beta deviations (%)**	0.00
**Clashscore**	2.4
**MolProbity score**	1.02

Supplementary Table 2 | [^{3}H]-NMS dissociation radioligand binding at mAChR WTs and mutants

	[^{3}H]-NMS dissociation rate (k_{off})[a]							pIC_{50}[b]
	10 µM Atropine	+ 10 µM Iperoxo	+ 30 µM LY2033298	+ 10 µM LY2033298	+ 10 µM Xanomeline	+ 30 µM Xanomeline	+ 100 µM Xanomeline	
**M_1 mAChR WT**	0.0182 ± 0.0007 (6)	0.0196 ± 0.0009 (6)	0.0032 ± 0.0003 (3)*	N.D.	0.0185 ± 0.0007 (6)	0.0169 ± 0.0008 (6)	0.0116 ± 0.0005 (6)*	3.75 ± 0.04 (6)
**M_2 mAChR WT**	0.055 ± 0.003 (10)	0.054 ± 0.003 (6)	0.0024 ± 0.0008 (6)*	N.D.	0.041 ± 0.003 (6)*	0.024 ± 0.003 (6)*	0.012 ± 0.002 (6)*	4.58 ± 0.03 (10)
**M_3 mAChR WT**	0.0089 ± 0.0005 (7)	0.0079 ± 0.0003 (7)	0.0019 ± 0.0002 (6)*	N.D.	0.0070 ± 0.0004 (7)*	0.0066 ± 0.0003 (7)*	0.0056 ± 0.0003 (7)*	3.74 ± 0.07 (7)
**M_4 mAChR WT**	0.0058 ± 0.0003 (14)	0.0063 ± 0.0003 (5)	0.0012 ± 0.0002 (7)*	0.0013 ± 0.0003 (4)*	0.0057 ± 0.0003 (6)	0.0045 ± 0.0003 (8)*	0.0032 ± 0.0002 (13)*	3.92 ± 0.03 (12)
**M_4 mAChR Y89A**	0.0041 ± 0.0002 (5)	N.D.	N.D.	*Can't calculate*	N.D.	N.D.	0.0004 ± 0.0002 (5)*	N.D.
**M_4 mAChR Y92A**	0.0049 ± 0.0003 (5)	N.D.	N.D.	0.0017 ± 0.0002 (4)*	N.D.	N.D.	0.0051 ± 0.0005 (5)	N.D.
**M_4 mAChR Q184A**	0.0063 ± 0.0007 (5)	N.D.	N.D.	0.0006 ± 0.0004 (4)*	N.D.	N.D.	0.0040 ± 0.0005 (5)*	N.D.
**M_4 mAChR F186A**	0.0053 ± 0.0004 (4)	0.0049 ± 0.0008 (3)	0.0046 ± 0.0006 (3)	N.D.	N.D.	0.0051 ± 0.0006 (3)	0.0051 ± 0.0006 (3)	N.D.
**M_4 mAChR L190F**	0.0059 ± 0.0005 (5)	N.D.	N.D.	0.0008 ± 0.0001 (4)*	N.D.	N.D.	0.0040 ± 0.0003 (5)*	N.D.
**M_4 mAChR W435A**	0.0028 ± 0.0004 (5)	N.D.	N.D.	0.0023 ± 0.0002 (4)	N.D.	N.D.	0.0029 ± 0.0004 (5)	N.D.
**M_5 mAChR WT**	0.0070 ± 0.0005 (5)	0.0059 ± 0.0004 (5)	0.0030 ± 0.0003 (4)*	N.D.	0.0049 ± 0.0004 (5)*	0.0053 ± 0.0005 (5)*	0.0034 ± 0.0004 (5)*	3.96 ± 0.09 (5)

Data represent the mean ± S.E.M. of (n) independent experiments performed in duplicate. N.D. not determined. * significantly different from 10 µM Atropine condition, $p < 0.05$, one-way ANOVA with Dunnett's multiple comparisons test.

[a] Specific binding data were fit to a single exponential decay to calculate the dissociation rate constants (k_{off} in min^{-1}).

[b] Dissociation rate constants of [^{3}H]-NMS in the presence of xanomeline were fit to a three-parameter logistic equation to derive a pIC_{50} inhibitory constant.
