# Supplementary material for: Xanomeline displays concomitant orthosteric and allosteric binding modes at the M4 mAChR
Source: Nat Commun. 2023 Sep 6;14:5440. doi: 10.1038/s41467-023-41199-5 (PMC10482975; doi:10.1038/s41467-023-41199-5)
Supplement: Supplementary file 3 — Reporting Summary [file 41467_2023_41199_MOESM3_ESM.pdf]

nature portfolio

Corresponding author(s): Ron O. Dror, Arthur Christopoulos, Celine Valant, David M. Thal

Last updated by author(s): Jul 10, 2023

Reporting Summary

Nature Portfolio wishes to improve the reproducibility of the work that we publish. This form provides structure for consistency and transparency in reporting. For further information on Nature Portfolio policies, see our Editorial Policies and the Editorial Policy Checklist.

Statistics

For all statistical analyses, confirm that the following items are present in the figure legend, table legend, main text, or Methods section.

n/a	Confirmed	
☐	☒	The exact sample size (*n*) for each experimental group/condition, given as a discrete number and unit of measurement
☐	☒	A statement on whether measurements were taken from distinct samples or whether the same sample was measured repeatedly
☐	☒	The statistical test(s) used AND whether they are one- or two-sided<br>*Only common tests should be described solely by name; describe more complex techniques in the Methods section.*
☒	☐	A description of all covariates tested
☐	☒	A description of any assumptions or corrections, such as tests of normality and adjustment for multiple comparisons
☐	☒	A full description of the statistical parameters including central tendency (e.g. means) or other basic estimates (e.g. regression coefficient) AND variation (e.g. standard deviation) or associated estimates of uncertainty (e.g. confidence intervals)
☐	☒	For null hypothesis testing, the test statistic (e.g. *F*, *t*, *r*) with confidence intervals, effect sizes, degrees of freedom and *P* value noted<br>*Give P values as exact values whenever suitable.*
☒	☐	For Bayesian analysis, information on the choice of priors and Markov chain Monte Carlo settings
☒	☐	For hierarchical and complex designs, identification of the appropriate level for tests and full reporting of outcomes
☒	☐	Estimates of effect sizes (e.g. Cohen's *d*, Pearson's *r*), indicating how they were calculated

Our web collection on statistics for biologists contains articles on many of the points above.

Software and code

Policy information about availability of computer code

Data collection	Data collection for cryo-EM data was conducted using SerialEM3.7.<br>Molecular dynamics (MD) systems were set up using the Schrodinger suite of software tools (Glide, Prime), version 2020-1, ParamChem server (v1), Dabble (2.7.6), Dowser (1999 release), and Gaussian (v16). MD trajectories were performed using the CUDA-enabled version of PMEMD in Amber18 and CPPTRAJ (v16).
Data analysis	For Cryo-EM and data analysis the following software was used: UCSF Chimera v1.16, UCSF ChimeraX v1.15, Relion v3.1, UCSF MotionCorr, CTFFIND 4.1.8, Coot v0.9, Phenix 1.20.1, PyMol v2.5, the GRADE web server, and MolProbity v4.<br><br>MD data analysis was carried out using the Visual Molecular Dynamics (VMD) Python interface(1.9.3), and visualized using the Matplotlib Python package(3.6.2). PyMOL(2.3.2) and VMD(1.9.3) were used for molecular visualization.<br><br>GraphPad Prism 9.02 was used for other data analysis and curve fitting.

For manuscripts utilizing custom algorithms or software that are central to the research but not yet described in published literature, software must be made available to editors and reviewers. We strongly encourage code deposition in a community repository (e.g. GitHub). See the Nature Portfolio guidelines for submitting code & software for further information.

Data

Policy information about availability of data

All manuscripts must include a data availability statement. This statement should provide the following information, where applicable:

- Accession codes, unique identifiers, or web links for publicly available datasets
- A description of any restrictions on data availability
- For clinical datasets or third party data, please ensure that the statement adheres to our policy

Atomic coordinates and cryo-EM maps for the reported structures were deposited in the Protein Data Bank under accession code 8FX5 (Human M4 muscarinic acetylcholine receptor complex with Gi1 and xanomeline) and in the Electron Microscopy Data Bank under accession code EMD-29524 (Human M4 muscarinic acetylcholine receptor complex with Gi1 and xanomeline). Previously published structures can be accessed via accession codes: 7TRS, TTRK, 7TRP, 7TRQ, 6OIJ, 6OIK, 6ZG4, 6ZFZ, 6ZG9, and 7V68. Simulation trajectories are available at https://doi.org/10.5281/zenodo.8136971. The source data underlying Figures 3 and 4 and Supplementary Figures 6 and 7 are provided as a Source Data file. The initial coordinate file, simulation input files, and trajectories will be available on Zenodo (10.5281/zenodo.8136971).

Research involving human participants, their data, or biological material

Policy information about studies with human participants or human data. See also policy information about sex, gender (identity/presentation), and sexual orientation and race, ethnicity and racism.

Reporting on sex and gender	N/A
Reporting on race, ethnicity, or other socially relevant groupings	N/A
Population characteristics	N/A
Recruitment	N/A
Ethics oversight	N/A

Note that full information on the approval of the study protocol must also be provided in the manuscript.

Field-specific reporting

Please select the one below that is the best fit for your research. If you are not sure, read the appropriate sections before making your selection.

[x] Life sciences   [ ] Behavioural & social sciences   [ ] Ecological, evolutionary & environmental sciences

For a reference copy of the document with all sections, see nature.com/documents/nr-reporting-summary-flat.pdf

Life sciences study design

All studies must disclose on these points even when the disclosure is negative.

Sample size	No statistical methods were used to predetermine sample size. All functional data were determined from at least three independent experiments. Five independent simulations were performed for each condition.
Data exclusions	For cryo-EM data analysis, micrographs with low estimated CTF resolution were excluded from processing. No other data were excluded.
Replication	For pharmacology assays more than three independent experiments were performed in duplicate. Pharmacology and MD simulations were reliably reproduced.
Randomization	This study was not a clinical trial or animal study that depends on randomization. Therefore, no randomization was attempted or needed.
Blinding	No group allocations were used or needed, therefore there was no blinding.

Reporting for specific materials, systems and methods

We require information from authors about some types of materials, experimental systems and methods used in many studies. Here, indicate whether each material, system or method listed is relevant to your study. If you are not sure if a list item applies to your research, read the appropriate section before selecting a response.

Materials & experimental systems

n/a	Involved in the study	
☐	☒	Antibodies
☐	☒	Eukaryotic cell lines
☒	☐	Palaeontology and archaeology
☒	☐	Animals and other organisms
☒	☐	Clinical data
☒	☐	Dual use research of concern
☒	☐	Plants

Methods

n/a	Involved in the study	
☒	☐	ChIP-seq
☒	☐	Flow cytometry
☒	☐	MRI-based neuroimaging

Antibodies

Antibodies used	Anti-FLAG M1 mouse polyclonal IgG2b was produced from a hydridoma obtained from ATCC (4E11).
Validation	Validated by manufacturer. Antibody was used only for purification of receptor complex.

Eukaryotic cell lines

Policy information about cell lines and Sex and Gender in Research

Cell line source(s)	Sf9 (Spodoptera frugiperda) and Tni (Trichoplusia ni) cells were purchased from Expression Systems; Flp-In CHO (Chinese Hamster Ovary) were purchased from Thermo Fisher Scientific.
Authentication	Cells were grown from original stocks purchased from suppliers. They were not authenticated in house.
Mycoplasma contamination	Cells were regularly tested to ensure there were free from mycoplasma contamination.
Commonly misidentified lines (See ICLAC register)	None used.
